# Language Skills in Children with Attention Deficit Hyperactivity Disorder and Developmental Language Disorder: A Systematic Review

**DOI:** 10.3390/children11010014

**Published:** 2023-12-21

**Authors:** Inmaculada Méndez-Freije, Débora Areces, Celestino Rodríguez

**Affiliations:** Department of Psychology, University of Oviedo, 33003 Oviedo, Spain; mendezinmaculada@uniovi.es (I.M.-F.); rodriguezcelestino@uniovi.es (C.R.)

**Keywords:** ADHD, DLD, idiopathic language impairment, neurodevelopmental disorders, comorbidity, language skills

## Abstract

(1) Background: Attention Deficit Hyperactivity Disorder (ADHD) and Developmental Language Disorder (DLD) are neurodevelopmental disorders that affect various areas of children’s development, such as language. It exists an emerging idea that ADHD is characterised by the presence of language difficulties, which can be explained by the high comorbidity between ADHD and DLD. The aim of this review is to analyse the empirical evidence of language competence in children aged 6–17 years old, diagnosed with ADHD and/or DLD. (2) Method: Fifteen studies with experimental designs were identified from Scopus, PsycINFO, and WoS databases, reporting on language skills in children diagnosed with ADHD and/or DLD. Studies relating executive functioning to language skills in this target population were also included. (3) Results: The literature is heterogeneous and different components of language are also examined. Even though the results are contradictory, they convincingly demonstrate there are overlapping symptoms between ADHD and DLD, such as language skills and executive functions. (4) Conclusions: The differences in the samples limit the generalisability of the results. Therefore, this review highlights the importance of considering language skills when designing individualised interventions for the population with ADHD and DLD, both in comorbidity and in isolation.

## 1. Introduction

Idiopathic language difficulties, i.e., difficulties in language development in the absence of an intellectual disability or other cause that might explain the difficulty, have been recognised since the early 19th century [1]. Although the terminology used has changed, in recent years the term Developmental Language Disorder (DLD) has come into use. About 7% of the population is affected by DLD, but the prevalence of DLD varies between studies and countries [2]. Although its prevalence is high compared to other neurodevelopmental disorders such as autism spectrum disorder (ASD) or childhood hearing loss, DLD is understudied [2,3,4]. Attention Deficit Hyperactivity Disorder (ADHD) and DLD are two of the most common neurodevelopmental and comorbid disorders [5,6]. Children with comorbid DLD and ADHD may show a wide variety of signs that may overlap and impact different areas, such as language and executive function.

Symptoms of comorbid DLD and ADHD vary in severity and can affect academic and social functioning, increasing interest in understanding these conditions to provide appropriate support and intervention.

### 1.1. Language Skills

Language skills enable an individual to use language at a level of accuracy that conveys meaning in production and comprehension. These skills include both productive and receptive language skills, such as speaking, listening, reading, and writing, and their effective use in a variety of practical contexts [7]. The five basic areas of language (phonology, morphology, syntax, semantics, and pragmatics) are essential for understanding and using language effectively. They work together to form a dynamic, integrated whole that enables individuals to communicate and comprehend spoken and written language [7,8].

Language skills are essential for communication and are crucial for navigating personal and professional life. Limited language skills affect a variety of areas, such as behaviours, acquiring knowledge, future occupation, social aspects, and well-being [9].

Language development plays an essential function in early stages of development and in overall cognitive and social progress. However, some children may face challenges in acquiring language skills, which can impact their academic performance and social interactions. Two common conditions that can affect language skills in children are ADHD and DLD [10].

### 1.2. Designations for Idiopathic Language Impairment

Idiopathic language impairment has been given a variety of names. Specific Language Impairment (SLI) was widely used in investigation articles to allude to the subset of children presenting language disturbances with unknown cause [11]. However, the term SLI was too restrictive, implying the child had a relatively pure language problem with no other impairments. For this reason, the term SLI was rejected by the CATALISE panel in 2017. Instead of SLI, an alternative terminology, Developmental Language Disorder (DLD), was proposed by the international CATALISE consortium and approved for employ in the cases where the origin of language impairment was unknow [12].

The CATALISE consortium also settles: the presence of neurobiological, environmental, or other risk factors does not avoid the diagnosis of DLD; other neurodevelopmental disorders may coexist with DLD (e.g., ADHD); and DLD diagnosis does not need a discrepancy between linguistic and non-verbal abilities [12].

Prior to the CATALISE consortium, the American Psychiatric Association (APA) published the Diagnostic and Statistical Manual of Mental Disorders, Fifth Edition [13], which established new guidelines for the diagnosis of language disorders. Instead of the term DLD, the DSM-5 manual adds the naming ‘language disorder’ within neurodevelopmental disorders to distinguish persevering troubles in using language.

The difference in terminology is a source of instability in establishing criteria for idiopathic language disorders. Following the CATALISE consortium, the term DLD will be used in this study.

Roughly 7.5% of the world’s population is affected by DLD. This diverse condition may have implications for using language, both at an expressive and receptive level. Furthermore, DLD may present different degrees of severity and impact various language domains (e.g., semantic, morphosyntactic, pragmatic) [14,15], which may explain the scarcity of proofs concerning the accuracy with which DLD diagnosis is made [16].

### 1.3. ADHD

Concurring to the fifth edition of the Diagnostic and Statistical Manual of Mental Disorders (DSM-5), ADHD is one of the most common neurodevelopmental disorders, affecting approximately 5–7% of the children and adolescents [13].

It is characterized by a persistent inattention, hyperactivity and/or impulsivity that interferes with function or development. ADHD symptoms can vary, and individuals may present primarily with inattention, hyperactivity, impulsivity, or a combination of both. These can lead to several difficulties such as emotional, behavioural, mental, and cognitive problems, and ADHD severity depends on the time of detection [13].

Though language challenges are not specifically mentioned as a fundamental diagnostic standard, children who have been diagnosed with ADHD exhibit significant deficiencies in their language skills across various aspects such as expressive, receptive, and pragmatic language modalities, resulting in overall language difficulties [17,18].

### 1.4. Comorbidity

According to the DSM-5, both ADHD and Language Disorder (LD) are considered neurodevelopmental disorders. These disorders can cause various difficulties such as emotional, behavioural, psychiatric, and cognitive problems, and their severity depends on the time of detection [13].

Some empirical reports suggest ADHD and DLD are not only the most common neurodevelopmental disorders, but may also be co-occurring disorders [5,6]. There is a complex relationship between DLD and ADHD, with overlapping and distinct features [4].

Scientific evidence supports the link between learning difficulties and executive functioning in neurodevelopmental disorders [19,20,21]. Children with comorbid DLD and ADHD may exhibit difficulties in executive functions, including working memory, cognitive flexibility, attention, and inhibitory control [17,22]. In this context, some studies have found the coexistence of these conditions had an additional effect on the educational results of children [23,24]. In fact, children with DLD may have attention deficits that are not significant enough to warrant a diagnosis of ADHD, but still affect their learning in the classroom [25].

Besides executive deficits, language difficulties are another overlapping symptom in comorbid DLD and ADHD. DLD is a well-defined condition characterized by impairments in receptive and expressive language, as evidenced by difficulty following multi-step instructions, difficulty with coherent conversation and expression, and impairments in reading and writing [26,27]

Overall, language problems are also observed in children with ADHD, although it is unclear which component of language is most affected. Some studies show difficulties at the morphosyntactic level [17], while others indicate significant impairments in language skills across expressive, receptive, and pragmatic domains [18,28].

Understanding the symptoms of comorbid DLD and ADHD, it is important to identify and address these difficulties to ensure appropriate diagnosis and treatment for affected children.

### 1.5. The Current Review

Research shows children diagnosed with ADHD have language impairments [6,17,29]. In fact, ADHD and DLD are two of the most common neurodevelopmental disorders and are co-occurring disorders [5,6], which increases the interest in comparing them.

Given the high prevalence rates of both ADHD and DLD [30], it is crucial to understand how these conditions affect children’s ability to use language. The purpose of this systematic review is to examine the current body of literature on language abilities in children with ADHD and/or DLD. By synthesising findings from different studies, we aim to identify the specific language difficulties experienced by these children and to explore potential overlaps or differences between the two conditions.

Understanding the language profiles of children with ADHD and/or DLD can have significant implications for diagnosis, intervention, and support. By identifying the specific language challenges these children face, clinicians, and educators can tailor interventions to address their unique needs. Additionally, this review will contribute to the existing literature by highlighting gaps in knowledge and suggesting avenues for future research.

In summary, this systematic review aims to provide a comprehensive overview of language skills in children with ADHD and/or DLD. By summarising the available evidence, we hope to improve our understanding of the language difficulties these children experience and inform effective strategies for assessment and intervention.

Finally, considering previous scientific literature has identified common symptoms in both ADHD and DLD, the hypothesis was the results would support the development of interventions that stimulate linguistic competencies (i.e., pragmatic skills, morphosyntactic, semantic abilities, …) as well as EF that usually tend to be impaired, particularly working memory. This is an innovative goal because it aims to identify data that support the need to improve language skills in children with ADHD and EF development in children with DLD.

## 2. Materials and Methods

The current systematic review was conducted using the Preferred Reporting Items for Systematic Reviews and Meta-Analyses (PRISMA) guidelines [31]. The PRISMA guidelines provide a 27-item checklist which allows reviewers to report the relevant information and make an accurate analysis of all the studies found. The objectives of this review are approved by the ethical committee of the University of Oviedo with the reference code “18_RRI_23” following PRISMA guidelines.

### 2.1. Search Strategy

The literature search was performed using three databases (Web of Science (WoS), PsycINFO and Scopus) in August 2023. The main objective was to analyse the empirical evidence of language competence in children and adolescents diagnosed with ADHD and/or DLD/SLI. The following key words were used: (“Specific Language Impairment” OR “Developmental Language Disorder” OR “DLD” or “SLI”) AND (“ADHD” OR “attention deficit hyperactivity disorder” OR “attention deficit-hyperactivity disorder” OR “attention deficit disorder”) AND (“language skills” OR “language” OR “language competence”) AND (“comorbid*” OR “co-occurring” OR “coexisting”).

### 2.2. Selection Criteria

The articles produced by the search were selected according to various inclusion/exclusion criteria, as follows.

Articles were selected if they fulfilled the described conditions:

(a) Empirical studies authored in the English or Spanish language.
(b) Included samples aged between 6 and 18 years.
(c) Empirical studies included children and youths diagnosed with ADHD and/or DLD/SLI.
(d) Published in the last ten years (2013–2023).
(e) Studies focusing on language skills and/or executive functioning in individuals diagnosed with ADHD and/or DLD/SLI.

On the other hand, articles were excluded if they met the following criteria:

(a) Conference papers, reviews, doctoral theses, meta-analyses, case reports or book chapters.
(b) Studies with a sample age range other than 6 to 18 years.
(c) Articles on neurodevelopmental disorders aside from DLD/SLI and/or ADHD, including cognitive delay, deafness, autism spectrum disorder, neurological deficits, or dyslexia.
(d) Studies focusing on biological variables, biomarkers, genetic variables, mental health, behavioural problems, clinical trials; studies focusing on parental reports.

### 2.3. Study Selection

**Figure 1:**
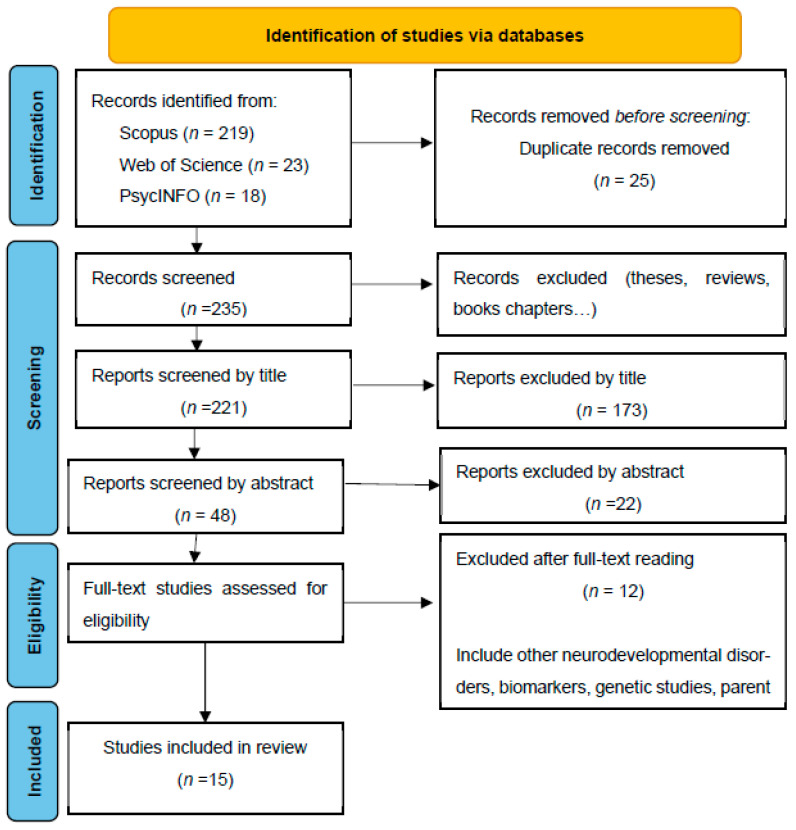
Flowchart of the article selection process.

A total of 260 research papers were selected in the reliable databases (219 from Scopus, 23 from WoS, and 18 from PsycINFO). Duplicates (25 results) and results from book chapters, doctoral theses, or conference papers (14 results) were removed. In total, 195 studies were excluded by title (*n* = 173) or by abstract (*n* = 22). Finally, 15 studies were selected as meeting the inclusion criteria. The whole process is illustrated in the following flowchart (Figure 1) in accordance with the PRISMA declaration [31].

## 3. Results

We first describe the general characteristics of the 15 selected studies (Table 1). Two-fifths (40%, *n* = 6) of the studies investigated the language skills of English speakers. The 15 studies were published in 12 different journals and, as Table 1 showed, the most studies were published in 2021.

Table 1 summarizes the demographic characteristics of the selected studies. The fifteen studies report results from nine countries with sample sizes varying from three hundred and seventy to five participants (Table 1). We selected studies with participants aged between 6 and 17 years, but in this case most studies (*n* = 9) focused on childhood (7–12 years). Only 40% (*n* = 6) of the selected studies reported participants’ gender. 

Just under half (46%, *n* = 7) of the articles studied populations with both a diagnosis of DLD and a diagnosis of ADHD. Only two of the fifteen studies included participants with comorbid DLD and ADHD, despite DLD and ADHD having high rates of comorbidity [30].

Ten of the selected articles compared the performance of different groups (ADHD group, DLD group, typically developing (TD) group) in different language tasks and the remaining five articles included measures of executive function in addition to language skills. There was a great deal of variability in the study of language skills. Some studies focused on narrative skills, while others focused on morphology, syntax, etc. (Table 2).

There was variation in the use of instruments, but the selected studies were consistent in using instruments such as the Children’s Communicative Checklist, Second Edition (CCC-2), the Clinical Evaluation of Language Fundamentals (CELF-4), or the Peabody Picture Vocabulary Test (PPVT-III). In terms of executive function, the most frequent items measured were working memory and attention (Table 2).

The evaluation of various language domains is an integral part of studying language skills. In our collection of articles, we discovered research concentrated on investigating language abilities in a group identified with DLD [31,35,37,39]. In these articles, there was a great diversity as they focused on different language domains, notably syntax, semantics, and pragmatics. For example, Kagnovich and colleagues [37] measured general linguistic aptitude and working memory and they found children with DLD had difficulties integrating visual information into long-term a-phonemic representations. However, it should be noted none of the selected studies addressed the study of phonology separately.

In the study conducted by Brinton and colleagues [32], the researchers analysed how five children with DLD expressed their internal thoughts and emotions when narrating a story based on pictures, as well as when they were directly questioned about these states. The explanations of the emotional states of the story characters’ emotions became more intense when prompted, but the children frequently used emotional words that did not precisely portray the events of the story.

Children with ADHD are known to have problems with language skills. In Vassiliu’s study [41], the performance of the ADHD group showed structural language difficulties, even though the ADHD group performed significantly better than the DLD group. As for pragmatics, the ADHD group performed worse than all other groups, but no statistical significance was found.

Similar results were found in the study of Staikova [40], who investigated pragmatic language functioning in children with ADHD using various formal tests and parent questionnaires. They found children with ADHD had less developed skills in several aspects of pragmatic language compared to their typically developing peers, and these problems were evident beyond general language tasks. 

Zenaro and colleagues [42] carried out a study comparing the oral narrative skills of children with ADHD and children with TD. In line with the aforementioned studies, the researchers discovered the ADHD group displayed reduced levels of coherence compared to the TD group. This was evident in their lower scores concerning the structural components of theme topic and outcome.

Narrative skills refer to the ability to tell a story or event in a coherent and organized manner. The literature suggests children with DLD may have problems with communication skills due to their impaired language function, whereas children with ADHD may have better grammar, general basic language skills, but still struggle with executive functions tasks [10]. Narrative tasks engage not only language skills but also executive functions [43]. Stanford and Delage [6,17] conducted a study on French speaking children, focusing on syntactic and morphosyntactic aspects, while simultaneously assessing executive-function variables. The EF and morphosyntactic profiles of children with ADHD and DLD are different [17]. Morphosyntax deficits are not specific to ADHD, but performance in the ADHD group may mimic morphosyntax impairment. This may explain the difference between the groups in the use of visual cues: children with DLD were more sensitive to visual cues than children with ADHD, which may require more attentional resources. In contrast, the DLD group was less sensitive to linguistic cues, as these cues required syntactic processing [6].

To determine which of the language areas is most affected in each of these disorders, and to understand the overlap of symptoms between the two, they need to be studied together. Our selection included six studies involving joint study of ADHD and DLD populations and a control group [6,17,29,32,36,41], while only two studies included samples with ADHD and DLD as comorbidity [22,34].

The findings are conflicting. In a study conducted by Paredes-Cartes and Moreno-García [29], notable distinctions in semantic and pragmatic abilities were observed between the DLD and ADHD groups. These results revealed children with DLD faced greater challenges in semantic language skills, whereas the ADHD group encountered more difficulties in pragmatic language skills. Helland and colleagues [36] aimed to investigate the linguistic characteristics of children with DLD. Their findings showed the DLD group experienced significantly greater difficulties than the ADHD group in both language and syntax scales. In terms of pragmatics, both groups faced similar challenges, while in the field of semantics, they showcased an equal degree of impairment.

Conflicting results have also been found in articles examining populations with comorbid ADHD and DLD, particularly in relation to the symptom of hyperactivity and language skills. Redmond and colleagues [22] focused on morphosyntactic characteristics of children with comorbid DLD and ADHD, as well as typically developing children, and those with only DLD. In the study, no noteworthy disparities were discovered between the groups with both DLD and ADHD. They additionally found there was a moderately positive correlation between the intensity of ADHD symptoms and the ability to recall sentences. In contrast, El Sady and colleagues [34] concluded hyperactivity had the strongest impact on language ability in individuals with ADHD. Specifically, the researchers found children with DLD and comorbid ADHD had poorer language reception compared to those with only DLD. 

Delgado and colleagues [33] compared the development of metapragmatic awareness in children with DLD, ADHD, and a control group. In the study, it was discovered there was a noticeable impact of age on children with ADHD. However, the DLD group and the control group showed significant differences. On the other hand, children who were diagnosed with predominantly inattentive ADD performed similarly to the control group.

Children with language impairment (LI) often perform worse on processing speed and working memory than their typically developing peers, and processing factors may contribute to an understanding of language disorders [44]. Ralli and colleagues [39] measured several EF variables, such as verbal fluency and working memory, as well as measures of expressive vocabulary and sentence completion. Their results indicated children with DLD were outperformed by their TD counterparts on measures of WM and verbal fluency (phonological and semantic).

Hyperactivity is also an important factor which affects language in ADHD [34]. However, O’Neil and colleagues [38] reported there was no significant association between the severity of pre-schoolers’ hyperactivity/impulsivity and their language skills at 4–6 years old.

The scientific research suggests processing speed is related to different aspects of language ability, including syntactic development, language impairment, social and language delays, and vocabulary/word learning [44,45].

Gooch and colleagues [35] conducted a longitudinal study in a British population to investigate the relationship between speed of processing (SOP), language and inattention/hyperactivity. The results showed the DLD group had increased symptoms of inattention/hyperactivity commonly associated with ADHD. Inattention/hyperactivity symptoms moderate the effect of SOP on language, but SOP does not predict subsequent language development.

After analyzing the results, in general terms, it was found children with ADHD had better general language skills, receptive and expressive language, and especially morphosyntactic skills than children with DLD. The main language problems in ADHD groups occur at the level of pragmatics and narrative coherence. It should be noted problems with language production and/or language comprehension are key features of DLD. Therefore, children with DLD would be expected to have more difficulties in these areas of language, especially semantics, morphosyntax, and structured language, than children with ADHD. Difficulties in the executive functioning of individuals with ADHD and DLD, such as impaired working memory and processing speed, may affect their pragmatic and structured language.

## 4. Discussion

The purpose of this research was to investigate the particular language challenges faced by children with DLD and/or ADHD and to investigate any potential overlaps or differences between the two disorders.

First, the review showed the symptoms of these two disorders overlap, including language difficulties and deficits in EF.

There is a great deal of variability in the research on the assessment of language skills. Conflicting results are to be expected due to the complexity of conducting assessments in different language domains and because it is important to be aware of the variability of the tests used in our selection of studies.

Some studies focused on receptive language, others on expressive language. The majority of the studies included in this review were concerned with examining grammar, semantics, and pragmatics, as these were the most impaired language areas in DLD and ADHD. However, the results are contradictory. Some research indicated children with DLD had more morphological and semantic difficulties than children with ADHD [6,17,29]. While other research also identified morphological and semantic difficulties in children with ADHD [41].

The studies included in the analysis presented diverse findings regarding the language performance of children with ADHD. Specifically, research focusing on language use among children with ADHD showed challenges in this area when compared to the control group [40], although the differences between the two groups were not consistently noteworthy [29]. On the other hand, positive correlations were occasionally discovered between symptoms of ADHD and tasks that require recalling sentences [22].

The study of language skills is a complex field involving several different factors. For example, the assessment of pragmatic language is challenging. Firstly, the construct encompasses both verbal and non-verbal skills. Furthermore, as the definition of pragmatics is linked to the social context, the assessment of pragmatic skills using standardised tests may not accurately reflect an individual’s actual ability to communicate in real-life situations [40]. In relation to narrative skills, structural elements are responsible for narrative coherence. These factors are important to build meaning for thematic maintenance and to present a coherent outcome related to the story problem situation that shows the relationship between the events being narrated [42]. They are also important in the ability to relate to internal situations, which include not only language deficits, but also limited social and emotional knowledge [32].

Another crucial aspect is to consider the relationship between EF and language competence, which are closely linked and interact in different ways [46]. In our selection of articles, only five included measures of EF in addition to language measures, which is particularly striking given the type of participants included in the studies (DLD and ADHD) would be expected to have deficits in EF.

It is generally interesting to consider socio-demographic variables, such as gender or age, as these may have an impact on the performance of the proposed tasks. Gender stereotypes may affect the diagnosis of girls with DLD and/or ADHD. There are gender differences in ADHD, with boys more likely to be diagnosed than girls. Despite similar symptoms, there is increasing evidence there are sex and gender differences in prevalence, presentation, expression, progression, and impairment. Girls with ADHD may experience greater functional impairment and have different long-term outcomes than boys with ADHD [47].

Gender also plays a role in DLD, with boys being at higher risk of the disorder and more likely to receive speech and language therapy services. A strong gender effect has been consistently reported: several studies have reported the consistent finding that males were more likely to experience language delay and DLD than females [48,49]. All of this shows how important it is to consider gender.

Finally, the age of the sample should be considered, as it may affect children’s language development. Children’s language skills develop throughout childhood, and the period of pregnancy at birth can also impact language development. In fact, children develop many of the oral language skills as they learn to read when they go to school, and their language skills continue to develop throughout early youth [50].

The ‘age effect’ also needs to be considered in the field of language impairment, especially at an early age. One example is the development of metapragmatic awareness happens recursively, in that as children grow older, they are not only able to identify the error between the context of the communicative act and the context of the communicative act and the linguistic expression, but also to explain it, thus achieving a reflexive control of language [33].

This metapragmatic awareness is expected to increase with age. In fact, adults with ADHD are acutely aware of how challenging social interaction situations are for them [10]. The executive-level deficits associated with ADHD lead to behavioural problems and pragmatic language difficulties [28]. Thus, without early intervention, difficulties at the pragmatic level are expected to persist into adulthood in people with ADHD. For individuals with DLD, difficulties at the structural level of language are expected to persist into adulthood due to deficits in processing speed and short-term memory [10,37,39]. At the group level, adults with DLD tend to have lower scores on language assessments than normative adults [51]. However, language development in DLD at the individual level is very heterogeneous: while some children grow up to be able to establish a valid pattern for social situations, others manifest permanent language difficulties in adulthood [10,52].

These findings highlight the need for longitudinal studies to examine language skills in the ADHD and/or DLD population to gain insight into the trajectories of language development [10].

Although language deficits in ADHD and DLD affect different components of language [29,40,41], language problems in both conditions may be caused by executive function deficits [10,17,39,46]. These language difficulties continue throughout adult life and increase with increasing environmental demands, leading to problems with academic success, learning problems, behavioural problems, and sometimes psychoemotional problems [10,24,26,51].

These results suggest there should be a standardized assessment tool that measured different language domains. It is important to assess students’ language abilities in a broad sense to identify children who may be at risk for language disorders early on. This tool should cover all aspects of language and help to identify children who may have these disorders.

## 5. Conclusions

This study has important implications for the field of language competences in DLD and/or ADHD. The results contribute to the understanding of language difficulties in these neurodevelopmental disorders by highlighting gaps in knowledge that may be addressed to improve the identification and intervention.

Intervention for ADHD and/or DLD should begin as early as possible to address cognitive and language problems. 

Regarding the ADHD interventions, based on the results of the tested studies, we can note in children with ADHD, along with the stimulation of executive functions, language skills should also be promoted. In this sense, pragmatic skills [40,41], as well as processes of syntactic coherence and text, as we have observed, were significantly reduced [29,36,42].

For its part, in case of children with DLD, it is important to provide targeted stimulation for language abilities, specifically in morphosyntactic [17] and lexical aspects [29,36]. Additionally, it is beneficial to focus on improving executive functioning associated with language such as WM and monitoring [35,39].

Medication intake in children with ADHD should be considered, because it can affect task performance [10]. But one of the studies selected for this review considered this factor.

Some of the limitations of the study are outlined below. The use of different terminology when talking about idiopathic speech problems makes it difficult to identify the research carried out. Similarly, the number of studies that included participants with comorbidity between DLD and ADHD was very small, even though they were two disorders that often co-occurred. Additionally, the limited number of studies and methodological diversity reduce the precision and generalizability of the findings. Hence, this review underscores the fact that further investigation is needed to clarify the relationship between language competence and these two neurodevelopmental disorders.

## Figures and Tables

**Table 1:** General characteristics of selected studies.

Author	Year	Journal	Country	Language
Brinton et al. [32]	2018	Communication Disorders Quarterly	USA	English
Delgado et al. [33]	2018	Pragmalingüística	Chile	Spanish
El Sady et al. [34]	2013	The Egyptian Journal of Medical Human Genetics	Egypt	Arabic
Gooch et al. [35]	2019	Child Development	UK	English
Helland et al. [36]	2014	Journal of Attention Disorders	Norway	Norwegian
Kaganovich et al. [37]	2021	Brain Sciences	USA	English
O’Neil et al. [38]	2016	Neuropsychology	USA	English
Paredes-Cartes y Moreno-Garcia [29]	2021	Revista Española de Pedagogía	Spain	Spanish
Ralli et al. [39]	2021	Brain Sciences	Greece	Greek
Redmond et al. [22]	2015	Language speech and hearing services in schools	USA	English
Staikova et al. [40]	2013	The Journal of Child Psychology and Psychiatry	USA	English
Stanford y Delage [6]	2021	Clinical Linguistics and Phonetics	Switzerland	French
Stanford y Delage [17]	2020	Frontiers in Psychology	Switzerland	French
Vassiliu et al. [41]	2022	Communication Disorders Quarterly	Greece	Greek
Zenaro et al. [42]	2019	Communication Disorders, Audiology and Swallowing	Brazil	Portuguese

**Table 2:** Summary of studies selected (*n* = 15).

Author(s)	Sample	Comorbidity Condition	Instrument(s)	Measures	Summary of Results
Brinton et al. (2018) [31]	Age range: 5–10 years Group(s): *n* =5 DLD Gender: 2 females/3 males	No	**Language skills:** Edmonton Narrative Norms Instrument (ENNI)	Narrative skills: internal response expressions in spontaneous and prompted condition.	Production and accuracy of internal plan Spontaneous condition: expressions were appropriate. Prompted condition: high production but the accuracy decreased. Production and accuracy of emotion words. Spontaneous condition: children describe few emotions. Prompted condition: greater variety of emotion words.
Delgado et al. (2018) [33]	Age range: 7–12 years Group(s): *n* = 6 DLD *n* = 6 ADHD *n* = 6 TD	No	**Language skills:** Metapragmatic Consciousness Assessment Test	Metapragmatic Awareness	TD group had the highest results. DLD group had the worst outcome. Age effect in the ADHD group. Significant discrepancies exist between the DLD group and the TD group.
El Sady et al. (2013) [34]	Age range: 3–6 years Group(s): *n* = 36 DLD + ADHD *n* = 25 DLD Gender: 9 females/27 males 9 females/16 males	Yes	**Language skills:** Language testing of Arabic speaking children	Receptive age Expressive age Semantic Syntax and phonology Pragmatics Receptive age quotient Expressive age quotient	Significant difference in the receptive age and the receptive age quotient: DLD + ADHD children had worse reception than DLD + no ADHD children. Hyperactivity was the most important factor affecting language in ADHD.
Gooch et al. (2019) [35]	Age range: 5–8 years Group(s): *n* = 129 DLD *n* = 370 TD	No	**Language skills:** Expressive One Word Picture Vocabulary Test (EOWPVT) Receptive One Word Picture Vocabulary Test (ROWPVT) School-Age Sentence Imitation Test-English 32 (SASIT-E32) Test for Reception of Grammar (TROG) Assessment of Comprehension and Expression (ACE) **Executive functions:** Rapid Automatized Naming (RAN) Visual Search (Apples Task) Coding (WPPSI-III) Simple Reaction Time	Vocabulary Grammar Narrative recall and comprehension Speed of processing:	Children with DLD perform worse on Speed of Processing (SOP) than their TD peers. The DLD group have elevated symptoms of inattention/hyperactivity, which are often associated with ADHD. Symptoms of inattention/hyperactivity moderate the effect of SOP on language, but SOP does not predict later language in formal schooling.
Helland et al. (2014) [36]	Age range: 6- 12 years Group(s): *n* = 19 DLD *n* = 21 ADHD *n* = 19 TD Gender: 2 females/17 males 3 females/21 males 2 females/17 males	No	**Language skills:** Children’s Communication Checklist–Second Edition (CCC-2)	Speech, syntax, semantics, coherence, inappropriate initiation, stereotyped language, use of context, nonverbal communication, social relations, interests.	Communication disorders were as severe in the ADHD group as in the DLD group. The ADHD group was as impaired as the DLD group on the scale measuring semantics. Language structure was more impaired in the DLD group. The ADHD group was more impaired on the interest scale.
Kaganovich et al. (2021) [37]	Age range: 7–13 years Group(s): *n* = 18 DLD *n* = 18 TD Gender: 5 females/13 males 7 females/11 males	No	**Language skills:** Photographic Expressive Language Test—2nd Edition (SPELT-II) Photographic Expressive Language Test—Preschool 2 (SPELT-P2) Core Language Score (Concepts and Following Directions, Recalling Sentences, Formulated, Sentences, Word Structure and Word Classes-2 Total (WC-2, 9–12-year-olds only), and Word Definitions (WD, 13-year-olds only). **Executive Functions:** Verbal working memory and nonword repetition test. Number Memory Forward. Number Memory Reversed subtests of the Test of Auditory Processing. Skills—3rd edition (TAPS-3).	General linguistic aptitude Working memory	Children with TD, but not children with DLD, can integrate visual information into long-term phonemic representations
O’Neil et al. (2016) [38]	Age range: 4–8 years Group(s): *n* = 90 ADHD *n* = 60 TD	No	Language domain of the NEPSY. Wechsler Individual Achievement Test, Second Edition (WIAT-II): Word Reading, Pseudoword, Decoding, Reading Comprehension, and Spelling subtest. Vanderbilt Assessment Scale, Teacher Informant: Reading and Written Expression performance in school.	Language (NEPSY) Reading Writing	At 4–6 years of age, there was no significant relationship between the severity of preschool children’s hyperactivity/impulsivity and their language skills. at 4–6 years. At 8 years, language ability determined the pathway from preschool inattention (but not hyperactivity/impulsivity).
Paredes-Cartes y Moreno-Garcia (2021) [29]	Age range: 7–12 years Group(s): *n* = 47 DLD *n* = 48 ADHD *n* = 47 TD Gender: 20 females/27 males 39 females/9 males 19 females/28 males	No	**Language skills**: The Objective and Criterion-referenced Language Suite (BLOC) The Peabody Picture Vocabulary Test (PPVT-III)	Language: morphology, syntax, semantics and pragmatics. Receptive vocabulary	Significant differences in semantic and pragmatic language skills were found between the three groups. Children with ADHD had fewer problems with semantic competence than children with DLD. Pragmatic competence: ADHD group had lower scores than the DLD group and the control group.
Ralli et al. (2021) [39]	Age range: 8 years Group(s): *n* = 29 DLD *n* = 29 TD Gender: 14 females/15 males 17 females/12 males	No	**Language skills:** Wechsler Intelligence Scale for Children-WISC III: vocabulary scale. Athena Test: the sentence completion subtest. **Executive functions:** - Working Memory Test Battery for Children. - n-back task. - Flanker task. - Borella’s task. - “How many—What number task” - Semantic fluency test. - Phonological fluency test.	Expressive vocabulary Sentence completion Working memory Updating Inhibition Switching Verbal fluency Phonological fluency	Children with DLD were outperformed by their TD peers on measures of WM capacity, updating, monitoring (mixing cost), and verbal fluency (phonological and semantic).
Redmond et al. (2015) [22]	Age range: 7–9 years Group(s): *n* = 19 DLD *n* = 19 ADHD + DLD *n* = 19 TD Gender: 10 females/9 males	Yes	**Language skills:** English Nonword Repetition Task. Test of Early Grammatical Impairment (TEGI).	Nonword repetition Sentence recall Tense marking	No significant differences were found between the ADHD and DLD + ADHD groups. A moderate positive correlation was found between the severity of ADHD symptoms and their ability to memorize sentences: children with higher levels of ADHD symptoms performed better than those with lower levels.
Staikova et al. (2013) [40]	Age range: 7–11 years Group(s): *n* = 28 ADHD *n* = 35 TD	No	**Language skills:** Children’s Communicative Checklist, Second Edition (CCC-2). Comprehensive Assessment of Spoken Language (CASL). Test of Pragmatic Language, Second Edition (TOPL-2) Narrative Assessment Profile: Discourse Analysis (NAP) Clinical Evaluation of Language Fundamentals, Fourth Edition (CELF-4)	Pragmatic language General language	Children with ADHD had poorer pragmatic language skills than their peers on all measures, even after controlling for general language skills.
Stanford and Delage (2021) [6]	Age range: 8 years Group(s): *n* = 20 DLD *n* = 20 ADHD *n* = TD	No	**Language skills:** Bilan Informatisé du Langage Oral (BILO) Phonological loop. **Executive functions:** Conners CBRS: inattention, hyperactivity.	Syntax selective attention, central executive, processing speed, attentional flexibility.	Children with DLD were more sensitive than children with ADHD to visual cues (DLD > ADHD), which were more implicit than the linguistic cues and may have required more attentional resources. For linguistic signals that require syntactic processing, the opposite pattern was true (ADHD > DLD).
Stanford and Delage (2020) [17]	Age range: 8 years Group(s): *n* = 20 DLD *n* = 20 ADHD *n* = 20 TD	No	**Language skills:** Bilan Informatisé du Langage Oral (BILO). Probe test. **Executive functions:** Sky Search task (TEA-ch) Digit recall task (WISC-IV) Opposite Worlds task (TEA-ch)	Morphosyntax Selective attention Working memory Attention shifting	Different EF and morphosyntactic profiles in children with ADHD and DLD. ADHD group: higher-order EF weakness and difficulty with the omnibus morphosyntax task. DLD group: lower- and higher-order limitations and struggled with both morphosyntax tasks. Deficits in morphosyntax are not characteristic of ADHD: their performance can mimic morphosyntactic impairment.
Vassiliu et al. (2022) [41]	Age range: 4–8 years Group(s): *n* = 25 DLD *n* =29 ADHD *n* = 29 TD Gender: 8 females/17 males 11 females/18 males 10 females/19 males	No	**Language skills:** Logometro tasks	Structural language Vocabulary: receptive and expressive Morphosyntax: expressive and receptive Pragmatic language	Children with ADHD faced difficulties with language skills and especially with structural language: they performed significantly lower than their TD peers but significantly higher than the DLD group. From a pragmatic perspective, ADHD children performed numerically worse than any other group, but no statistical significance was found.
Zenaro et al. (2019) [42]	Age range: 6–10 years Group(s): *n* = 20 ADHD *n* = 20 TD Gender: 6 females/14 males	No	**Language skills:** Frog, Where are you?	Narrative skills	The ADHD group scored lower scores on the structural elements of “theme/topic” and “outcome” and a narrative with a lower degree of coherence than the TD group.

Note. DLD = Developmental Language Disorder; ADHD = Attentional Deficit Hyperactivity Disorder; TD = Typical Development.
